# Fine-tuning protein language models to understand the functional impact of missense variants

**DOI:** 10.1016/j.csbj.2025.05.022

**Published:** 2025-05-28

**Authors:** Ali Saadat, Jacques Fellay

**Affiliations:** a School of Life Sciences, Ecole Polytechnique Fédérale de Lausanne, Lausanne, Switzerland; b Swiss Institute of Bioinformatics, Lausanne, Switzerland; c Precision Medicine Unit, Biomedical Data Science Center, Lausanne University Hospital and University of Lausanne, Lausanne, Switzerland

**Keywords:** Missense variant, Mechanistic interpretation, Protein language models, Fine-tuning, Token classification

## Abstract

Elucidating the functional effects of missense variants is crucial yet challenging. To investigate their impact, we fine-tuned protein language models, including ESM2 and ProtT5, to classify 20 protein features at amino acid resolution. In addition, we trained a fully connected neural network classifier on frozen embeddings and compared its performance to fine-tuning in order to quantify the added value of task-specific adaptation. We then used the fine-tuned models to: 1) identify protein features enriched in either pathogenic or benign missense variants, and 2) compare the predicted feature profiles of proteins with reference and alternate alleles to understand how missense variants affect protein functionality. We show that our models can be used to reclassify variants of uncertain significance and provide mechanistic insights into the functional consequences of missense mutations.

## 1 Introduction

Recent advancements in sequencing technologies and bioinformatic analyses have significantly enhanced their utility in clinical settings, enabling more precise and comprehensive genetic diagnostics [1]. This progress has led to the generation of vast amounts of clinical-grade, personal genetic data, providing unprecedented opportunities to uncover the genetic basis of diseases. However, this surge in data also brings substantial challenges, particularly in the interpretation of variants of uncertain significance (VUS). Many putatively deleterious variants identified in the coding regions of the genome are missense variants, which can alter protein function by substituting one amino acid for another [2]. Accurately determining the clinical significance and functional impact of these variants remains a formidable task [3].

In the context of diagnostic genetic testing, the American College of Medical Genetics and Genomics (ACMG) and the Association for Molecular Pathology (AMP) guidelines are widely employed for sequence variant interpretation [4]. These guidelines propose a standardized framework that integrates diverse types of evidence, including population data [2], [5], computational predictions [6], [7], and functional studies. One of the criteria in these guidelines, PM1 (moderate evidence of pathogenicity), considers whether a missense variant is located in a mutational hotspot or a critical, well-established functional domain (e.g., the active site of an enzyme) that lacks benign variation [4]. While this criterion provides valuable guidance, identifying such regions poses significant challenges. It requires robust annotation of functional domains and systematic quantification of pathogenic and benign enrichment [8].

Previous studies have attempted to address this challenge using gene-disease databases such as ClinVar [9], which aggregates clinical interpretations of genetic variants, in combination with population frequency data from resources like gnomAD [2], a large-scale database of allele frequencies across diverse human populations. These efforts have identified regions enriched with pathogenic variants and provided insights into specific proteins [10], [11], [12]. Here, pathogenicity enrichment refers to the statistical overrepresentation of pathogenic variants compared to benign variants in specific annotated features. However, these analyses have been limited by the incomplete annotation of the human proteome, leaving many proteins and functional regions unexplored. Furthermore, little attention has been paid to understanding the mechanistic impact of missense variants on specific protein features, which could provide deeper insights into their pathogenicity.

To address these gaps, we harness the power of protein language models (PLMs), including ESM2 [13] and ProtT5 [14], for variant classification and interpretation. ESM2 is based on an encoder-only transformer architecture, while ProtT5 adopts an encoder-decoder design; both have demonstrated remarkable potential in capturing structural and functional properties of proteins through pretraining on large-scale protein sequence data. Recent studies have demonstrated the efficacy of fine-tuning these PLMs for a variety of downstream tasks [15], [16], [17]. Fine-tuning refers to updating pretrained model weights on a specific supervised task to adapt the model's representations [18]. Compared to traditional computational approaches, fine-tuned PLMs do not require multiple sequence alignments or handcrafted input features, making them highly scalable and broadly applicable to any protein sequence [19].

**Fig. 1:**
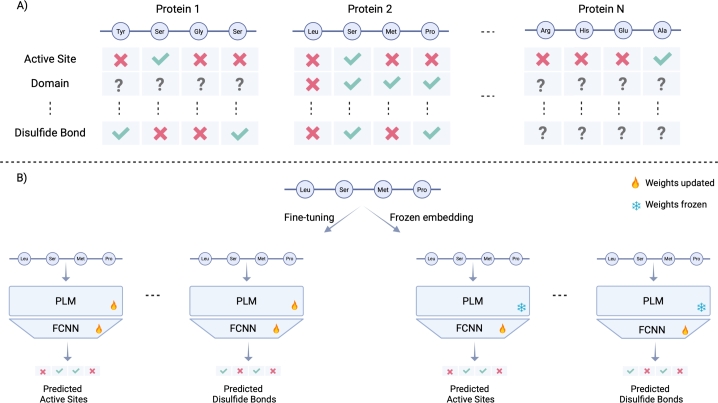
A) Overview of the dataset: 20,434 human protein sequences were downloaded from UniProtKB/Swiss-Prot. Each sequence was annotated with 20 features at amino acid resolution. B) Comparison of fine-tuning and frozen embedding classifiers for amino acid-level classification: For each feature, we trained two models to predict its presence or absence at each amino acid. One approach involved fine-tuning a pretrained PLM, while the other used a frozen embedding classifier, implemented as a fully connected neural network (FCNN) trained on top of the frozen PLM embeddings. This resulted in 20 fine-tuned models and 20 frozen embedding classifiers per PLM. Figure created with BioRender.com.

**Fig. 2:**
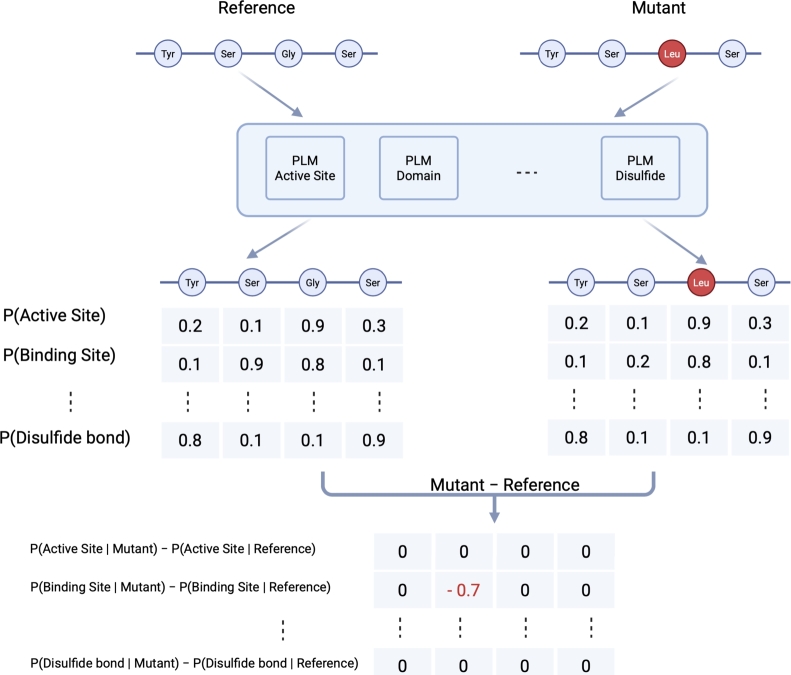
Mechanistic variant interpretation using fine-tuned PLMs: To assess the impact of a missense variant, both the reference and alternate protein sequences are analyzed using fine-tuned PLMs. The difference in predicted probabilities between the alternate and reference sequences is computed for each feature. These probability shifts can indicate a gain or loss of functional or structural features caused by the mutation. Figure created with BioRender.com.

In this study, we fine-tuned ESM2 and ProtT5 to predict diverse protein features at amino acid resolution (Fig. 1), using a token classification setup in which each amino acid (token) is labeled based on the presence or absence of specific features. To fine-tune PLMs efficiently, we applied Low-Rank Adaptation (LoRA) [20], a parameter-efficient method that inserts trainable, rank-decomposed matrices into the model's architecture while keeping most pretrained weights frozen [21]. This strategy substantially reduces the number of trainable parameters, enabling faster training and lower memory usage while preserving performance. In addition to fine-tuning, we trained a frozen embedding classifier, consisting of a fully connected neural network, on top of the frozen embeddings generated by the pretrained model. This setup allows us to evaluate the quality of learned representations before updating the PLM weights. By comparing the performance of the frozen embedding classifier with that of the fine-tuned model, we quantified the added value of task-specific adaptation. We then used the fine-tuned models to: 1) identify protein features enriched in either pathogenic or benign variants, thereby highlighting critical functional regions, and 2) compare reference and alternate protein sequences to understand how missense variants affect protein functionality (Fig. 2).

To clarify how our approach differs from prior methods, we highlight key distinctions in purpose and application. Methods like AlphaMissense [22] and DeepSequence [23] are designed to predict a pathogenicity score to classify missense variants as pathogenic or benign. QBind [17], on the other hand, fine-tunes ESM2 to predict binding residues at the amino acid level, an objective similar to one of our models, but focuses solely on prediction performance. In contrast, our approach leverages fine-tuned PLMs not just for feature prediction, but for downstream applications such as mechanistic variant interpretation and functional reannotation. Given a variant, our models allow us to infer *why* it might be pathogenic by identifying gain or loss of structural or functional features, providing mechanistic context that complements existing tools, enhances interpretability, and supports more informed variant prioritization and clinical decision-making.

We demonstrate the practical application of our framework by reclassifying VUS in the gnomAD database [2]. By integrating our feature-based approach, we also provide protein- and feature-specific insights into how missense variants influence protein structure and function. This work not only contributes to improving variant interpretation but also offers a framework for leveraging PLMs to elucidate the functional impact of genetic variation.

## 2 Methods

### 2.1 Data collection and splitting

We selected 20,434 human proteins from UniProtKB/Swiss-Prot [24] and extracted their amino acid sequence as well as their protein family membership. We annotated the proteins with 20 features including:

• Functional features: active site, binding site, and DNA binding site
• Sub-cellular location: topological domain and trans-membrane
• Post-transcriptional modification (PTM) and processing: disulfide bond, modified residue, propeptide, signal peptide, and transit peptide
• Structure: *β*-strand, *α*-helix, and turn
• family and domain: coiled coil, compositional bias, domain, motif, region, repeat, and zinc finger

To create train, validation, and test splits, we clustered all protein sequences using MMseqs2 [25] with thresholds of 20% coverage and 20% sequence identity. This resulted in 7,538 unique clusters, which we used to assign proteins into 70% training, 15% validation, and 15% testing sets, ensuring minimal data leakage by performing the splits at the cluster level.

### 2.2 Fine-tuning, frozen embedding classifiers, and evaluation

For each feature, we fine-tuned ESM2 at five model sizes (8 million, 35 million, 150 million, 650 million, and 3 billion parameters), as well as ProtT5, to classify amino acids based on the presence or absence of the feature (Table 1). A fully connected neural network was added on top of the PLM encoder to perform per-residue binary classification. This architecture was used consistently across all features and model sizes to ensure comparability. To fine-tune the models efficiently, we used LoRA (Low-Rank Adaptation) [20], a parameter-efficient technique that inserts rank-decomposed trainable matrices into attention layers while keeping most pretrained weights frozen [21].

**Table 1:** Summary of protein language models used in this study. Emb: embedding, M: million.

Model	Architecture	#Params (encoder)	#Layers	Emb. size	HuggingFace ID
ProtT5	Encoder-Decoder	1200M	24	1024	prot_t5_xl_uniref50
ESM2-8M	Encoder	8M	6	320	esm2_t6_8M_UR50D
ESM2-35M	Encoder	35M	12	480	esm2_t12_35M_UR50D
ESM2-150M	Encoder	150M	30	640	esm2_t30_150M_UR50D
ESM2-650M	Encoder	650M	33	1280	esm2_t33_650M_UR50D
ESM2-3B	Encoder	3000M	36	2560	esm2_t36_3B_UR50D

In addition to fine-tuning, we also trained a frozen embedding classifier, consisting of a fully connected neural network applied to the frozen pretrained embeddings. This approach enables evaluation of the quality of the learned representations before updating the model weights. To quantify the added benefit of task-specific adaptation, we compared the performance of the frozen embedding classifier and the fine-tuned model using AUROC. We also report macro-averaged F1 score, precision, recall, Matthews Correlation Coefficient (MCC), and accuracy.

We did not perform hyperparameter tuning due to computational costs. Instead, we adopted training settings informed by recent benchmarking from Schmirler et al. [16], which included two token classification tasks. Specifically, we used a hidden size of 32 for the classification head. For LoRA, we set the rank to 4, α=1, and applied it to the query, key, value, and output projections of the attention layers. All models were trained with a learning rate of $3\times 10^{-4}$ and a dropout rate of 0.2, using cross-entropy loss.

Each model was trained for 10 epochs, and the checkpoint with the lowest validation loss was selected for evaluation. Training was performed using a single Nvidia A100 GPU (40 GB memory). Inference requires significantly less memory than training, as also noted by Schmirler et al. [16]; thus, models can be run on more modest hardware, such as Nvidia A10G GPUs (24 GB memory).

### 2.3 Protein annotation inference

For each feature, we extracted the amino acid sequences from all proteins that lacked information about that feature. We utilized the corresponding fine-tuned model to predict presence or absence of the feature at each amino acid. To check the quality of predictions, we compared the distribution of GERP (Genomic Evolutionary Rate Profiling) conservation scores [26] and REVEL (Rare Exome Variant Ensemble Learner) pathogenicity scores [7] between labeled and predicted amino acids. GERP scores quantify evolutionary constraint by measuring rejected substitutions; higher scores indicate stronger conservation. REVEL is an ensemble method that predicts missense variant pathogenicity based on multiple tools and features.

### 2.4 Applications

• Variant reclassification: according to the ACMG/AMP guidelines [4], missense variants that are located in a mutational hot spot and/or critical functional domains are more likely to be pathogenic (moderate evidence of pathogenicity, PM1). To identify such regions, we obtained 46,504 missense pathogenic and 53,169 missense benign variants from ClinVar [9] (variants with conflicting classification were removed). We also extracted 18,991 non-redundant missense variants with minor-allele frequency ≥ 0.02 from gnomAD [2], and added them to the set of benign variants (they are considered benign due to high frequency in population). We performed two-sided Fisher's exact test to identify protein features that are significantly enriched in pathogenic or benign variants. After detecting regions with enrichment of pathogenic variants, we used them to reclassify variants of unkown significance (VUS) in gnomAD. To do so, we extracted all missense variants from gnomAD, and assigned a probability of pathogenicity (PoP) without using PM1. Then we focused on VUS and calculated a new PoP score by adding the PM1 evidence which is applied for missense variants located in regions with high enrichment of pathogenic variants. Finally, we calculated the fraction of VUS that were reclassified by adding PM1.
• Variant interpretation: To understand the potential impact of a missense variant on protein function, we designed a workflow to provide mechanistic insight into how the variant may alter specific protein features (Fig. 2). Briefly, we input both the reference and mutant protein sequences into the fine-tuned PLMs, and then compute the difference in predicted feature probabilities between the two. A gain or loss of a particular feature is detected if the predicted label changes between the reference and mutant sequences. Additionally, a threshold can be applied to the absolute value of the differential score to identify only those changes exceeding a defined magnitude. We performed a sensitivity analysis using thresholds ranging from 0.1 to 0.9 in steps of 0.1 to examine how the choice of threshold affects the number of predicted feature changes that pass the cutoff. This framework enables prediction of feature-level changes at amino acid resolution and may inform the design of follow-up functional experiments. To demonstrate this application, we obtained 6,974 curated missense variants across 107 genes from ClinGen [27], retaining only genes with at least one pathogenic and one benign variant. We then applied the variant interpretation workflow (Fig. 2) to all selected variants.

## 3 Results

### 3.1 Fine-tuning, frozen embedding classifiers, and evaluation

We retrieved amino acid sequences of 20,434 human proteins from UniProtKB/Swiss-Prot [24], along with associated annotations. The number of annotated human proteins per feature in UniProtKB/Swiss-Prot is shown in Fig. S1, and Fig. S2 displays the number and percentage of labeled amino acids per feature, highlighting the label imbalance across features. To create train, validation, and test splits, we clustered all protein sequences using MMseqs2 [25] with thresholds of 20% coverage and 20% sequence identity, resulting in 7,538 unique clusters. For each feature, the annotated proteins were divided into 70% training, 15% validation, and 15% testing sets. This cluster-based splitting strategy minimizes sequence similarity between training and test sets, thus promoting generalization and simulating prediction on unseen protein sequences.

**Fig. 3:**
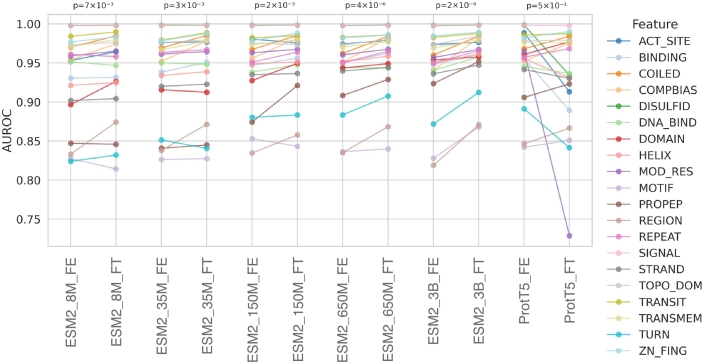
Performance of fine-tuned (FT) vs. frozen embedding (FE) classifier models: Models were evaluated using feature-specific test splits. The vertical axis indicates the AUROC value. P-values displayed above the plot were calculated using a paired Wilcoxon test.

We performed fine-tuning and trained frozen embedding classifiers on ProtT5 as well as ESM2 at five model sizes: 8 million, 35 million, 150 million, 650 million, and 3 billion parameters. For each protein feature, the training split was used to fine-tune the PLM or to train a frozen embedding classifier for amino acid-level classification, while the validation split was used to select the checkpoint with the lowest validation loss. The performance of both approaches was then evaluated on feature-specific test sets to quantify the performance gain or loss associated with fine-tuning. As shown in Fig. 3, most model–feature combinations exhibited improved performance with fine-tuning compared to the frozen embedding classifiers, although in some cases performance was unchanged or slightly reduced, consistent with previous findings [16]. Based on these results, we selected the fine-tuned ESM2-3B model for subsequent analyses. Fig. S3 presents the $F_{1}$, precision, recall, MCC, AUROC, and accuracy of ESM2-3B across all features.

Since larger protein families may be better represented in the original training data of ESM2, their strong performance could potentially mask lower performance in smaller, less-characterized families. To investigate this, we grouped the proteins in the test set by protein family and computed the average performance of the ESM2-3B model for each group. As shown in Fig. S4, no systematic trend was observed between family size and performance.

### 3.2 Protein annotation inference

**Fig. 4:**
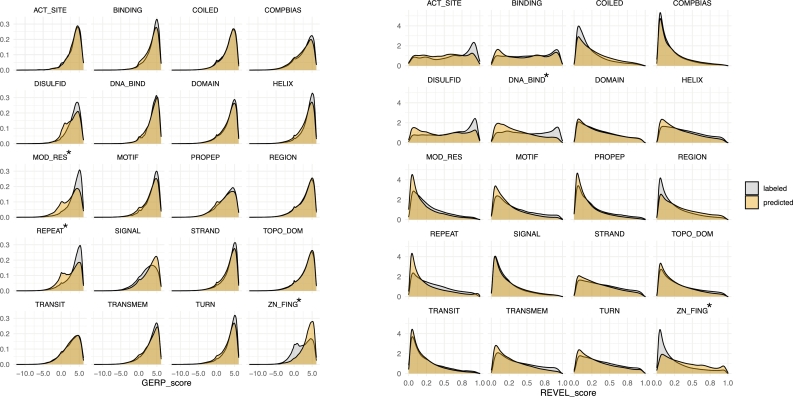
Comparison of characteristics between predicted and labeled amino acids: The left panel shows the distribution of conservation scores, while the right panel displays pathogenicity scores for labeled and predicted amino acids across all features. Features marked with an asterisk (*) indicate significant differences (p-value ≤ 0.05 and effect size ≥ 0.2) based on the Kolmogorov-Smirnov test.

We utilized fine-tuned ESM2-3B models to predict the presence or absence of features in proteins lacking annotations. The number of labeled and predicted proteins, as well as amino acids, is detailed in Fig. S5. To evaluate prediction quality, we analyzed the distribution of conservation scores (GERP [26]) and variant pathogenicity scores (REVEL [7]) between labeled and predicted amino acids (Fig. 4). Using the Kolmogorov-Smirnov test [28] to compare distributions per feature, we found that most features showed no significant differences. Notable exceptions included DNA binding sites and zinc fingers for REVEL scores, as well as modified residues, repeats, and zinc fingers for GERP scores.

### 3.3 Applications

#### 1. Variant classification

**Fig. 5:**
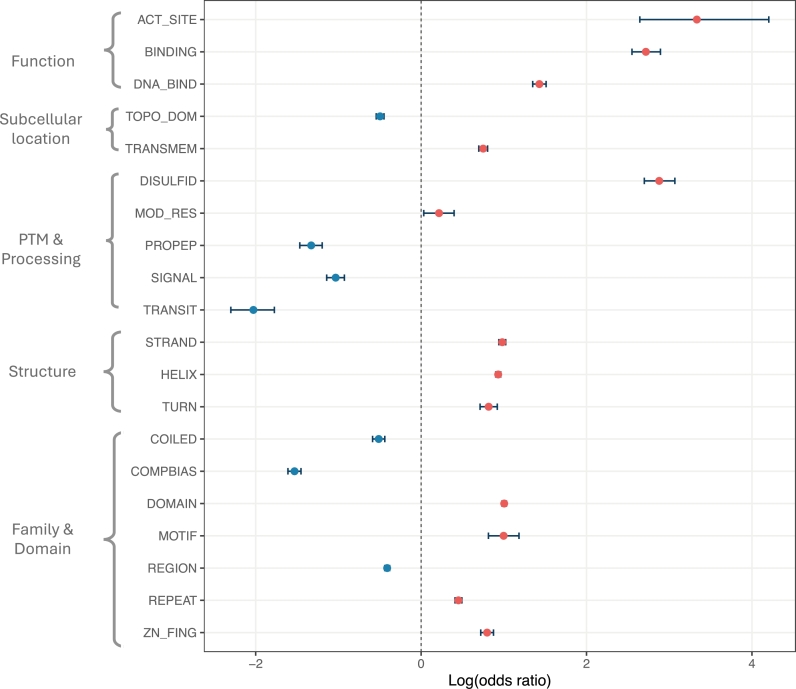
Enrichment of features in pathogenic and benign variants: A two-sided Fisher's exact test was conducted for each feature using pathogenic variants from ClinVar and benign variants from ClinVar/gnomAD. The odds ratio (OR) was calculated as $OR=\frac{a\cdot d}{b\cdot c}$, where *a* and *b* are the number of pathogenic and benign variants, respectively, within a given feature, and *c* and *d* are the number of pathogenic and benign variants outside the feature. Thirteen features were significantly enriched in pathogenic variants (red dots), while seven features were enriched in benign variants (blue dots).

Using 46,504 pathogenic variants from ClinVar and 72,150 benign variants from ClinVar/gnomAD, we performed two-sided Fisher's exact tests to identify protein features significantly associated with pathogenic or benign variants. Fig. 5 highlights 13 features enriched in pathogenic variants, including active site, binding site, DNA binding site, transmembrane regions, disulfide bonds, modified residue, *β*-strands, *α*-helices, turns, domains, motifs, repeats, and zinc fingers. Leveraging these 13 features, we aimed to reclassify variants of uncertain significance (VUS) in gnomAD.

To detect VUS, we applied ACMG/AMP criteria (see Appendix A) and calculated a probability of pathogenicity (PoP) for all missense variants in gnomAD. A total of 1,692,568 variants with 0.1< PoP <0.9 were classified as VUS. We then refined the PoP score by incorporating PM1 evidence, applied to missense variants located in the 13 protein features with significant enrichment of pathogenic variants. This refinement led to the reclassification of 110,304 (6.5%) VUS as pathogenic.

#### 2. Variant interpretation

We identified 771 curated missense variants across 54 genes from ClinGen [27], each gene containing at least one pathogenic and one benign variant. To assess the potential impact of these variants, we applied the workflow described in Fig. 2. Briefly, we passed the reference and alternate protein sequences through the fine-tuned ESM2-3B model and computed the difference in predicted feature probabilities at each amino acid position.

**Fig. 6:**
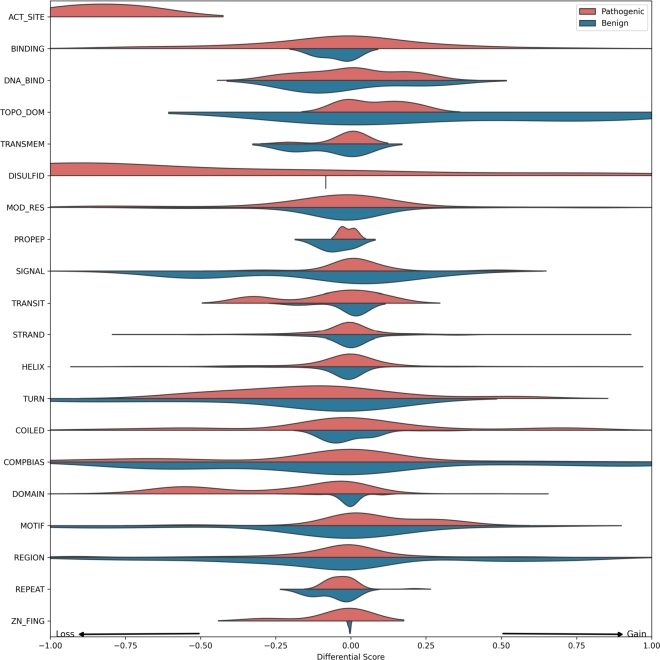
Predicted impact of pathogenic and benign missense variants on protein features: A total of 771 curated variants across 54 genes, each with at least one pathogenic and one benign missense variant, were identified from ClinGen. The variant interpretation workflow (Fig. 2) was applied individually to each variant, and the differences in predicted probabilities between the alternate and wild-type proteins were recorded.

We observed that pathogenic missense variants frequently disrupted critical features, particularly active sites, disulfide bonds, and functional domains (Fig. 6). In contrast, benign variants had a much milder effect, most commonly involving changes in more tolerable features such as compositional bias and signal peptides, consistent with our earlier enrichment analysis (Fig. 5).

**Fig. 7:**
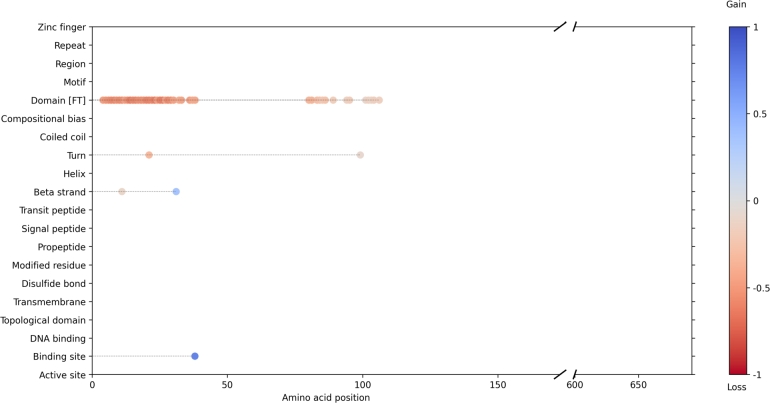
Predicted impact of the p.His35Asp variant on *DCLRE1C* using the variant interpretation workflow (Fig. 2). Each dot represents a predicted loss or gain of a feature at the corresponding amino acid position.

**Fig. 8:**
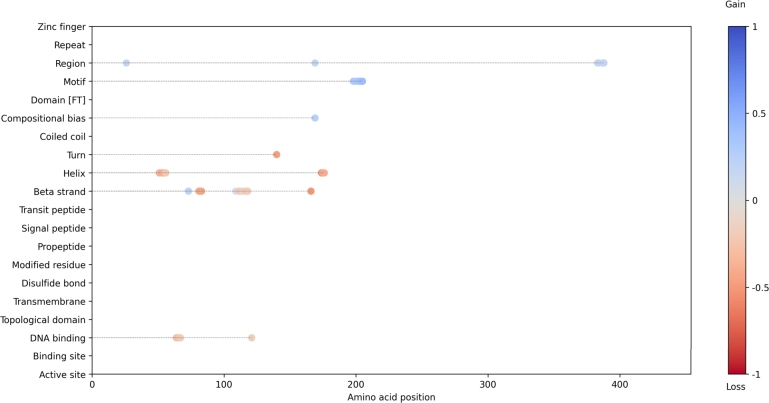
Predicted impact of the p.Ala134Pro. variant on *RUNX1* using the variant interpretation workflow (Fig. 2). Each dot represents a predicted loss or gain of a feature at the corresponding amino acid position.

To further demonstrate a practical application of our interpretability approach, we highlight two pathogenic variants as examples: *DCLRE1C*:p.His35Asp and *RUNX1*:p.Ala134Pro. The *DCLRE1C* variant led to the loss of domain annotations at multiple positions, the gain of a binding site, and changes in structural features including turns and *β*-strands, as well as additional binding sites (Fig. 7). The *RUNX1* variant was associated with alterations in structural features such as turns, *β*-strands, and *α*-helices, along with changes in functional annotations including region, motif, compositional bias, and DNA binding (Fig. 8).

When applying the variant interpretation framework, a threshold can be used to include only feature changes with large probability shifts. To assess the impact of threshold selection, we performed a sensitivity analysis using values ranging from 0.1 to 0.9. As expected and shown in Fig. S6, lower thresholds resulted in a greater number of predicted feature changes, reflecting increased sensitivity, while higher thresholds produced fewer but more confident changes, indicating greater specificity. Although no threshold was applied in the illustrative examples above, one can be used in practice to adjust the trade-off between sensitivity and specificity based on the requirements of the downstream application.

## 4 Discussion

This study introduces a novel application of protein language models (PLMs) to deepen our understanding of the functional consequences of missense variants. By fine-tuning PLMs on specific protein features, we developed a robust and accessible toolset capable of classifying and interpreting missense variants with remarkable precision and granularity.

Compared to traditional approaches such as evolutionary conservation metrics or models relying on multiple sequence alignments, fine-tuned PLMs offer several advantages. They learn directly from primary sequence data and can generalize across the entire proteome without the need for handcrafted features or alignments. Fine-tuning enables task-specific adaptation and supports the interpretation of mechanistic variants, something that black-box pathogenicity scores alone cannot achieve.

Using these fine-tuned models, we quantified the enrichment of pathogenic variants across a broad set of protein features and successfully reclassified 6.5% of variants of uncertain significance (VUS) in gnomAD as pathogenic. In addition, our models predicted the structural and functional consequences of specific missense variants, as illustrated by the *DCLRE1C*:p.His35Asp and *RUNX1*:p.Ala134Pro examples in Fig. 7, Fig. 8, respectively. These case studies demonstrate how our interpretive framework can identify disrupted features at the residue level.

A key contribution of our work is the advancement of interpretability in variant effect prediction. Many state-of-the-art models, including AlphaMissense [22] and DeepSequence [23], produce accurate predictions but operate as black boxes, limiting their utility in experimental design and clinical settings [29]. This lack of transparency can undermine trust and hinder follow-up efforts by biologists and clinicians who need actionable insights. Our approach directly addresses this gap by providing mechanistic interpretations of variant effects. Rather than predicting the pathogenicity score, our models indicate which protein features, such as domains, active sites, or structural motifs, are likely to be affected. This enables users to reason about the biological basis of pathogenicity and design focused experiments or therapeutic interventions. Such interpretive capability complements existing classifiers and provides a foundation for evidence-based variant reclassification and functional follow-up.

The ability to detect gain or loss of specific structural and functional features at amino acid resolution has significant biological and clinical implications. For example, identifying the loss of an active site or a disulfide bond upon mutation offers a concrete mechanistic explanation for protein dysfunction that can be experimentally validated. This layer of interpretation also aids in prioritizing variants for validation or therapeutic targeting. In clinical genetics, our models may support variant reclassification under ACMG/AMP guidelines, particularly for VUS located in functionally enriched regions. By offering interpretable evidence of functional disruption, our framework has the potential to complement existing predictors and improve diagnostic decision-making.

Despite these advances, several limitations remain. While our models perform well in annotated protein regions, predictions in poorly characterized or unannotated regions require further validation. Regarding variant datasets, ClinVar [9] provides high-quality, clinically curated data but is biased toward well-studied genes. In contrast, gnomAD [2] offers broader population coverage but still overrepresents individuals of European ancestry. Additionally, although training a multi-task model to predict all 20 features jointly could theoretically leverage shared signal (e.g., between binding sites and domains), this was not feasible due to annotation sparsity. We therefore chose to fine-tune separate models for each feature to maximize the use of available labels.

This work underscores the growing impact of protein language models in both research and clinical genomics. By bridging predictive accuracy with mechanistic interpretability, our approach offers a powerful means to uncover the biological consequences of missense variants. As large-scale genomic data continues to expand, such interpretable and accessible tools will be essential for translating sequence-level information into functional insights and improving the diagnosis and management of genetic diseases [30], [29], [31], [32], [33].

## Declaration of generative AI and AI-assisted technologies in the writing process

During the preparation of this work, the authors used GPT-4 in order to improve writing and readability.

## CRediT authorship contribution statement

**Ali Saadat:** Writing – review & editing, Writing – original draft, Visualization, Methodology, Investigation, Formal analysis, Data curation, Conceptualization. **Jacques Fellay:** Writing – review & editing, Supervision, Project administration, Funding acquisition, Conceptualization.

## Declaration of Competing Interest

The authors declare no competing interests.

## Data Availability

The code for this study is available at: https://github.com/AliSaadatV/ESM2-Missense-Impact-Analysis. Data, metrics, and weights for fine-tuned and frozen-embedding models are available at: https://zenodo.org/records/15441302. Lead contact: Requests for further information and resources should be directed to and will be fulfilled by the lead contact, Jacques Fellay (jacques.fellay@epfl.ch).

## Appendix Supplementary material

MMC Supplementary figures as well as ACMG/AMP guidelines summary. MMC
