# Supplementary material for: Fine-tuning protein language models to understand the functional impact of missense variants
Source: Comput Struct Biotechnol J. 2025 May 28;27:2199–207. doi: 10.1016/j.csbj.2025.05.022 (PMC12166733; doi:10.1016/j.csbj.2025.05.022)
Supplement: MMC — Supplementary figures as well as ACMG/AMP guidelines summary. [file mmc1.pdf]

5 Supplementary figures 474

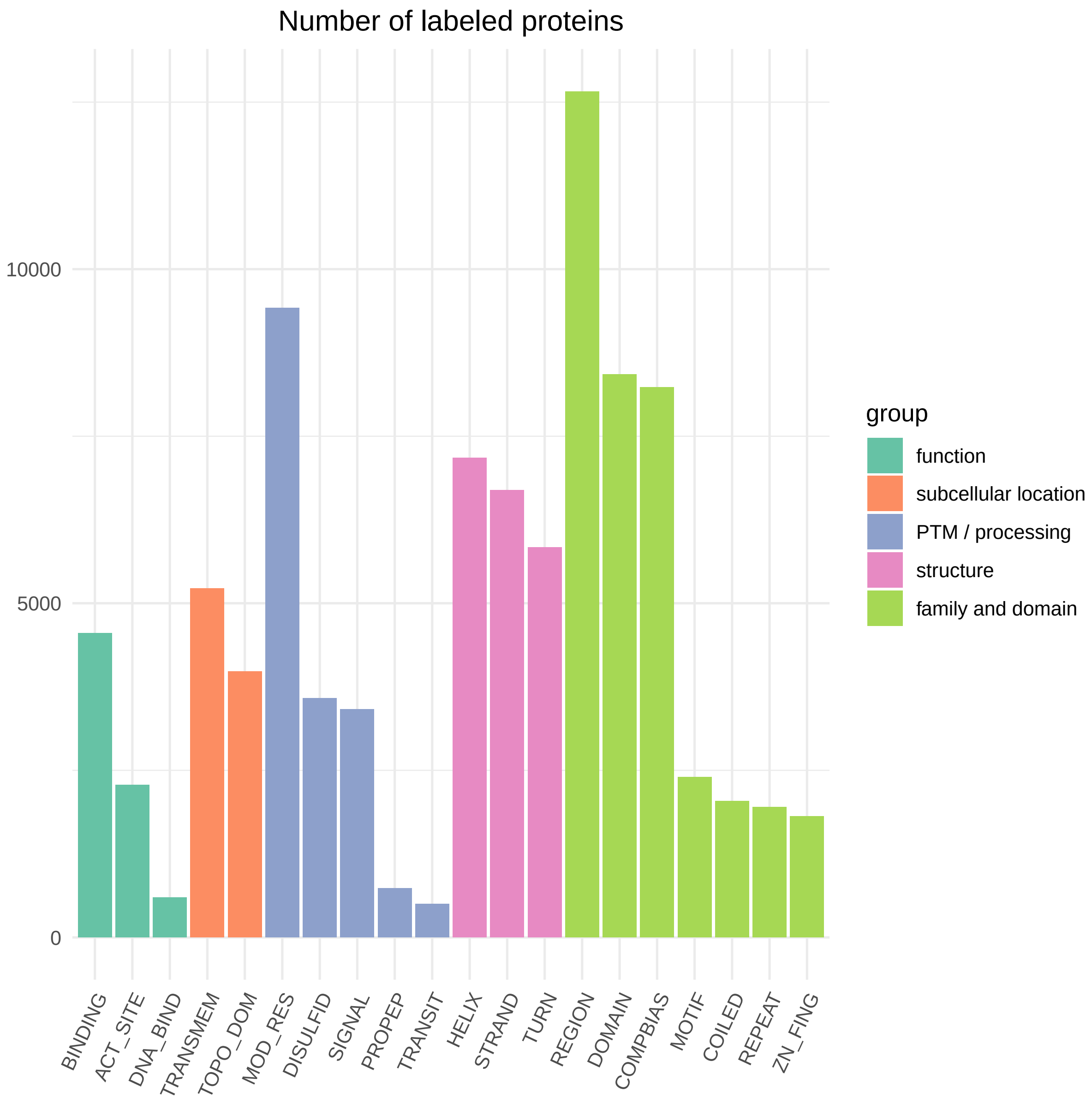

Supplementary Figure S1: Number of annotated protein per feature in UniProtKB/Swiss-Prot.

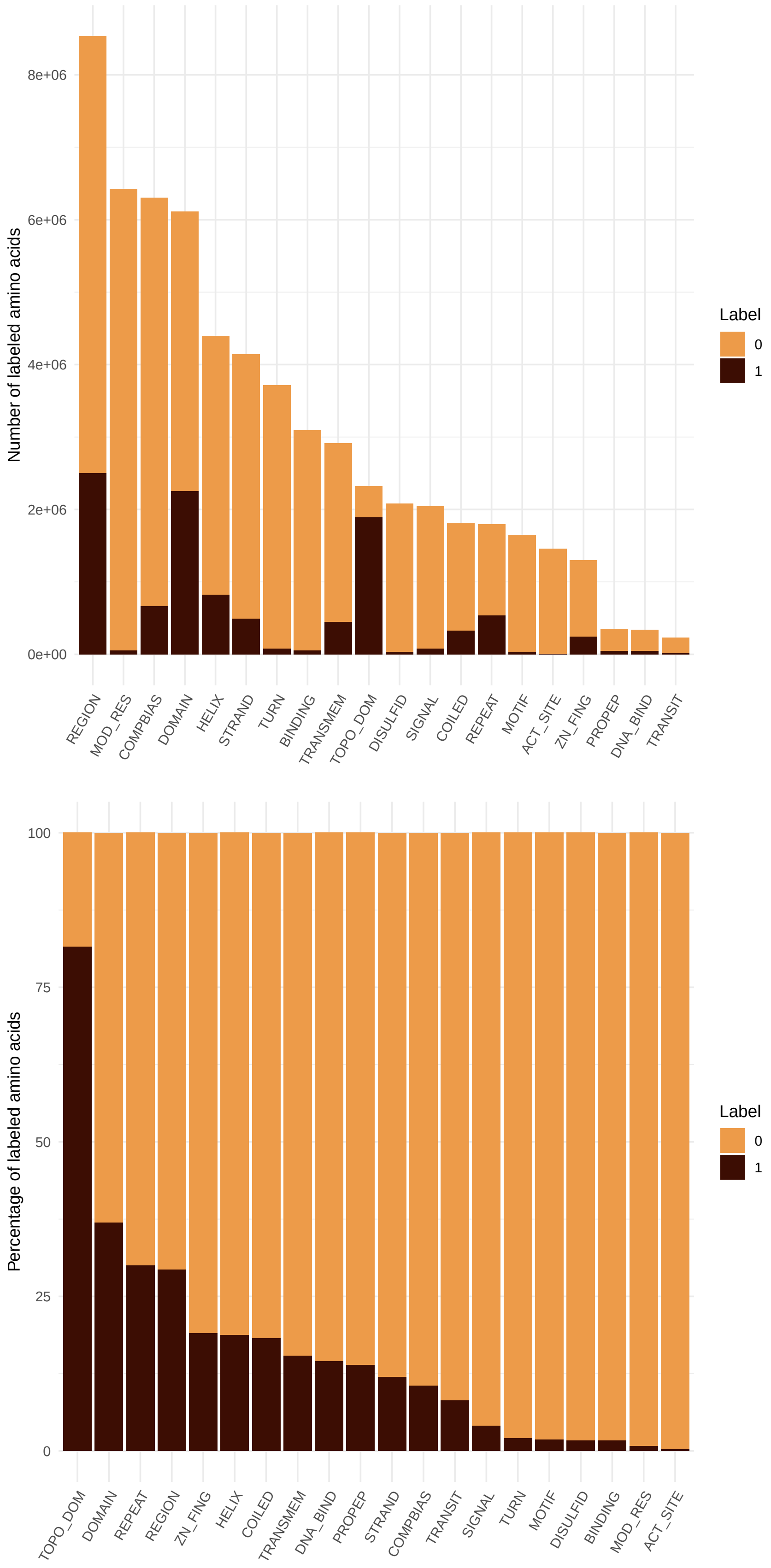

Supplementary Figure S2: Number (top panel) and percentage (bottom panel) of annotated amino acids per feature in UniProtKB/Swiss-Prot.

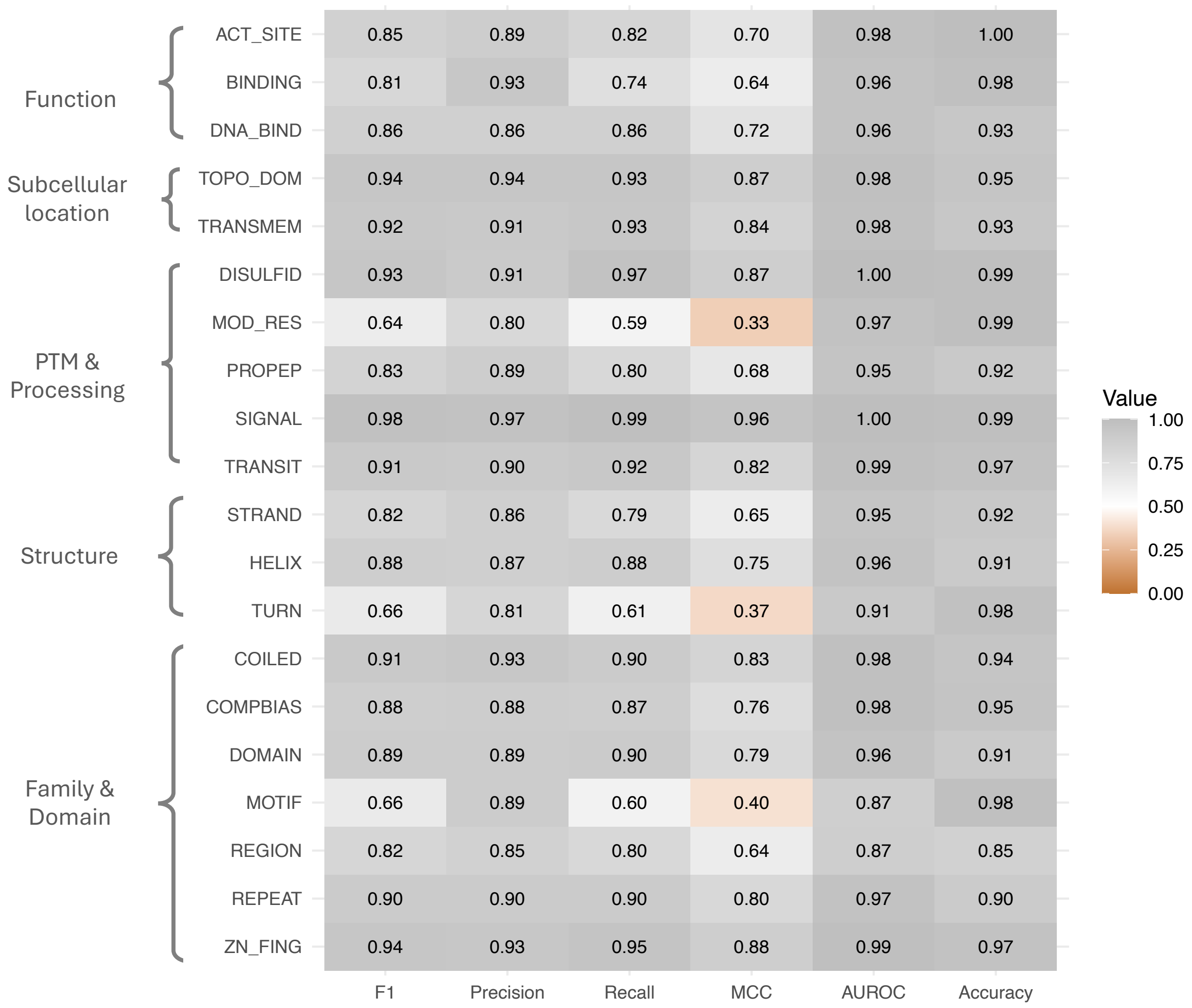

Supplementary Figure S3: F_1, precision, recall, MCC, AUROC, and Accuracy of the fine-tuned ESM-3B model.

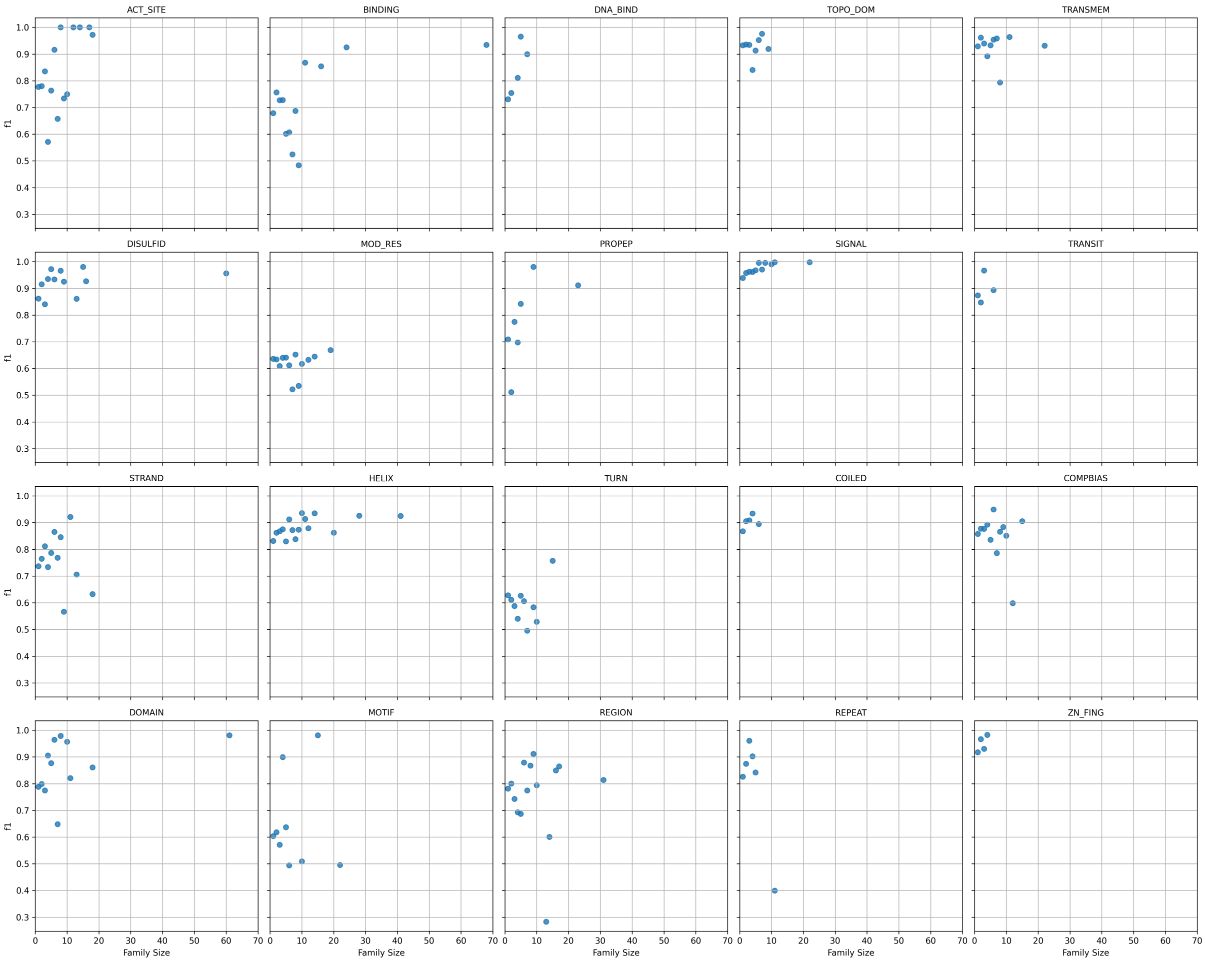

Supplementary Figure S4: Average F_1 score of ESM2-3B on the test set, stratified by protein family size.

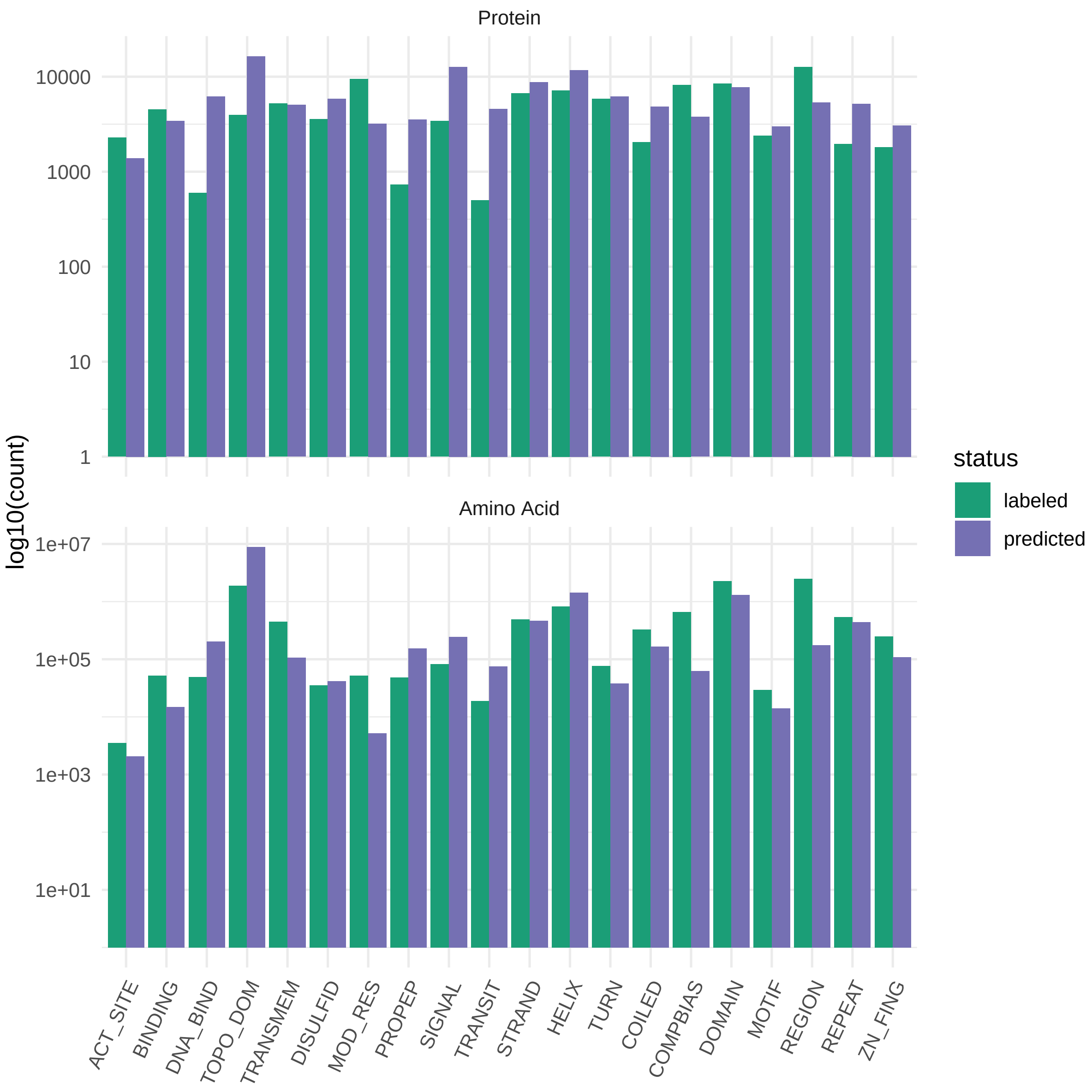

Supplementary Figure S5: Number of predicted and labeled proteins and amino acids.

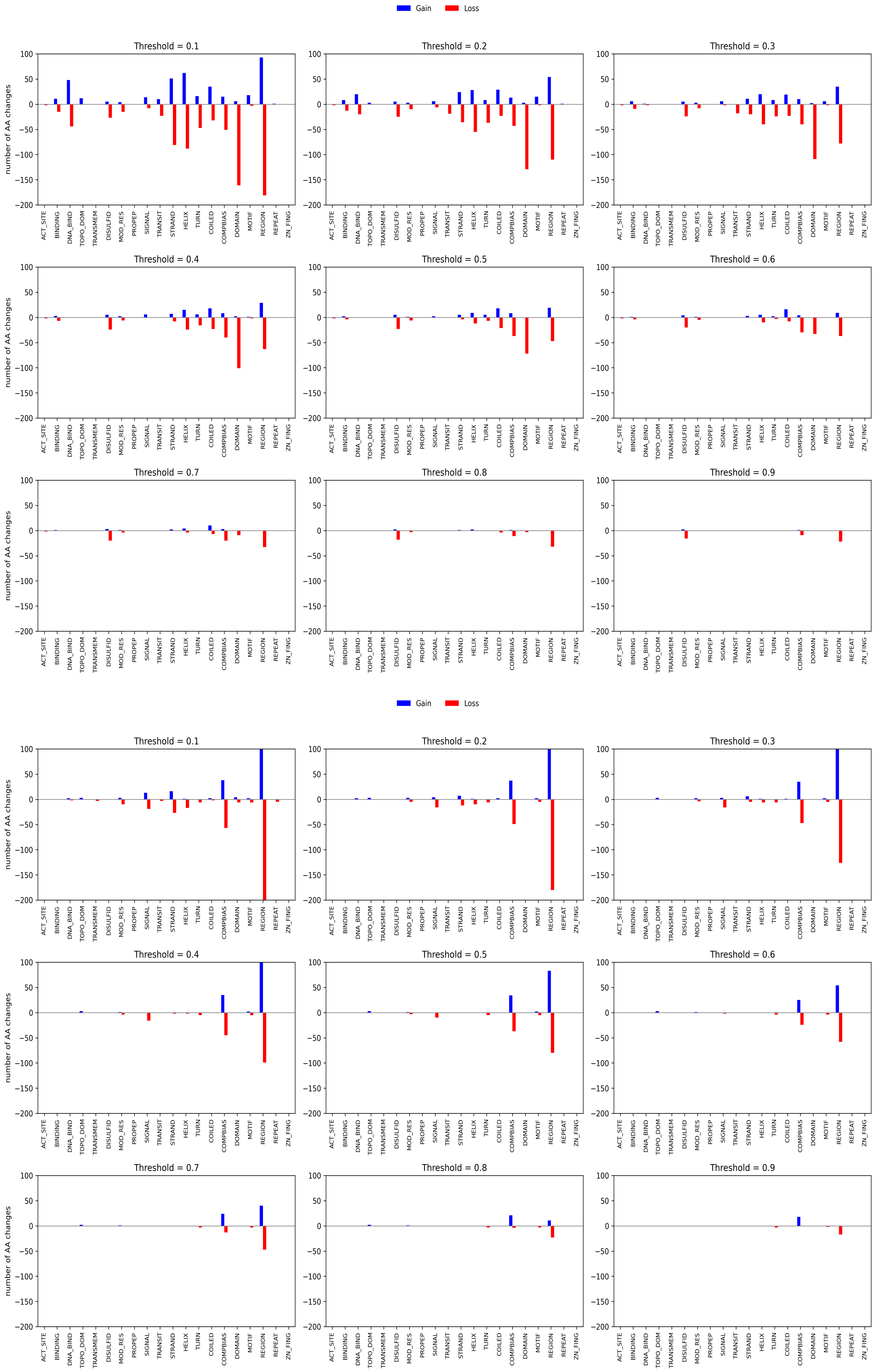

Supplementary Figure S6: Effect of threshold selection on the number of predicted amino acid feature changes due to missense variant. The top three rows show results for pathogenic variants, while the bottom three rows correspond to benign variants.

A Appendix

Probability of pathogenicity (PoP) calculation

We used ACMG/AMP guidelines[4] to classify the variants into putative pathogenicity groups, as described in our previous work[34]. In summary, we gather all the available evidences for a variant. Figure S7 summarizes all the ACMG/AMP criteria that we used.

	Benign		Pathogenic		
	Strong	Supporting	Supporting	Moderate	Strong
Population and controls	MAF is high in the population (BS1)			MAF is rare in the population (PM2)	
Computational		MIssense in a gene where mostly truncating variants cause disease (BP1)<br><br>Computational evidence suggest no impact on gene product (BP4)	Computational evidence supports a deleterious effect on the gene product (PP3)	Novel missense change at an amino acid residue Where a different pathogenic missense change has been seen before (PM5)	Same amino acid change as an established pathogenic variant (PS1)
Functional	Well-established functional studies show no deleterious effect (BS3)		Missense in a gene with low rate of benign missense variants and missense variants are common mechanism of disease (PP2)	Mutational hotspot or well-studied functional domain without benign variation (PM1)	Well-established functional studies show a deleterious effect (PS3)

Supplementary Figure S7: the summary of ACMG/AMP criteria used for variant classification. MAF: minor allele frequency

To calculate the probability of pathogenicity (PoP), we use the Bayesian framework developed by Tavtigian et al.[35]. For a given variant, the PoP is calculated as follow:

$$P_x = \text{number of pathogenic criteria applied at the level of } x$$
$$x \in \{\text{Strong}, \text{Moderate}, \text{Supporting}\}$$

$$B_y = \text{number of benign criteria applied at the level of } y$$
$$y \in \{\text{Strong}, \text{Supporting}\}$$

$$\text{odds of pathogenicity (OP)} = 350^{\left(\frac{P_{\text{Strong}}}{2} + \frac{P_{\text{Moderate}}}{4} + \frac{P_{\text{Supporting}}}{8} - \frac{B_{\text{Strong}}}{2} - \frac{B_{\text{Supporting}}}{8}\right)}$$

$$\text{probability of pathogenicity (PoP)} = \frac{OP \times 0.1}{((OP - 1) \times 0.1 + 1)}$$
